# Adjunctive Use of Locally Delivered Statins in Periodontal Therapy and Pre‐Implant Bone Regeneration: A Systematic Review and Meta‐Analysis

**DOI:** 10.1002/cre2.70364

**Published:** 2026-04-28

**Authors:** Koki Yoshida, Sofia Heller, Nur Mousa, Lovisa Olén, Farah Asa'ad

**Affiliations:** ^1^ Division of Oral Medicine and Pathology, Department of Human Biology and Pathophysiology, School of Dentistry Health Sciences University of Hokkaido Hokkaido Japan; ^2^ Institute of Odontology The Sahlgrenska Academy at University of Gothenburg Göteborg Sweden; ^3^ Department of Oral Biochemistry, Institute of Odontology The Sahlgrenska Academy at University of Gothenburg Göteborg Sweden

**Keywords:** bone regeneration, local drug delivery, periodontal therapy, statins

## Abstract

**Objectives:**

This systematic review and meta‐analysis aimed to evaluate the adjunctive beneficial effects of locally delivered statins on probing pocket depth (PPD), clinical attachment level (CAL), bleeding on probing (BoP), and radiographic bone outcomes in both periodontal therapy (Step 2 non‐surgical periodontal therapy and Step 3 periodontal surgery) and pre‐implant bone regeneration procedures.

**Methods:**

The review followed the PRISMA 2020 statement and was registered in PROSPERO (CRD420251105739). PubMed and Scopus were searched through July 2025 for randomized controlled trials (RCTs) evaluating the adjunctive use of locally delivered atorvastatin (ATV), rosuvastatin (RSV), or simvastatin (SIM) in periodontal therapy and pre‐implant regenerative procedures. Risk of bias was assessed using the Cochrane Risk of Bias 2 tool (RoB 2). Random‐effects meta‐analysis was conducted when extractable data were available at commonly reported follow‐ups (6 months for Step 2; 9 months for Step 3).

**Results:**

Twenty‐one RCTs were included (13 Step 2, 6 Step 3, 2 pre‐implant bone regeneration). In Step 2 therapy, adjunctive statins improved clinical outcomes compared with scaling and root planing alone, with pooled mean differences (MDs) of approximately 1.4–2.3 mm for PPD and 1.7–2.2 mm for CAL. Numerically larger pooled effects were observed for SIM; however, no direct head‐to‐head comparisons between statins were performed, and heterogeneity was high. In Step 3 therapy, three trials contributed to 9‐month pooling, showing smaller but significant benefits (PPD MD 0.80 mm; CAL MD 0.69 mm), with moderate heterogeneity. Pre‐implant bone regeneration trials were clinically heterogeneous and showed inconsistent radiographic outcomes, precluding quantitative synthesis.

**Conclusions:**

Locally delivered statins provide clinically relevant adjunctive benefits in Step 2 periodontal therapy and modest additional improvements in Step 3 therapy, particularly when incorporated into regenerative protocols. Evidence for pre‐implant bone regeneration remains limited and heterogeneous. Further multicenter RCTs with standardized clinical and radiographic outcomes are needed.

**Abbreviations**
ALN: alendronate
ATV: atorvastatin
BBM: bovine bone mineral
BL: baseline
BMP: bone morphogenetic protein
BoP: bleeding on probing
CAL: clinical attachment level
COI: conflict of interest
EDTA: ethylenediaminetetraacetic acid
GI: gingival index
HA: hydroxyapatite
IBDs: intrabony defects
MCL: methylcellulose
MD: mean difference
MF: metformin
N/A: not available
NSPT: non‐surgical periodontal therapy
OM: occlusive membrane
PBM: perforated barrier membrane
PI: plaque index
PMT: periodontal maintenance therapy
PPD: probing pocket depth
PPPM: polypropylene membrane
PR: papilla reflection
PRF: platelet‐rich fibrin
RCTs: randomized controlled trials
REML: restricted maximum likelihood
RoB: risk of bias
RSV: rosuvastatin
SIM: simvastatin
SPC: supportive periodontal care
SRP: scaling and root planing

## 1 Introduction

Periodontitis is a chronic inflammatory disease characterized by the progressive destruction of the tooth‐supporting tissues (Kwon et al. 2021). It is among the most prevalent oral diseases worldwide and a leading cause of tooth loss in adults (Wu et al. 2025). Standard periodontal therapies, including Step 2 subgingival instrumentation/non‐surgical periodontal therapy (NSPT) and Step 3 periodontal surgery, aim primarily to control inflammation and eliminate infection. Step 3 therapy may include regenerative procedures applied to selected defect types. Although regenerative therapies have demonstrated measurable clinical benefits, their outcomes remain variable, partly due to differences in defect morphology and patient‐specific healing capacity, highlighting the need for adjunctive agents that may enhance healing and regenerative outcomes (Avila‐Ortiz et al. 2022; Herrera et al. 2022; Sanz et al. 2020).

Statins, a class of 3‐hydroxy‐3‐methylglutaryl‐CoA (HMG‐CoA) reductase inhibitors commonly prescribed for lipid lowering, also exert pleiotropic effects relevant to periodontal regeneration, including anti‐inflammatory and bone anabolic effects (Tahamtan et al. 2020; Kommuri et al. 2025; Wang and Xu 2025). They modulate osteoclast and osteoblast activity, enhance bone morphogenetic protein (BMP)‐2 expression, and improve endothelial function, collectively supporting their potential role in periodontal therapy and pre‐implant bone regeneration (Tahamtan et al. 2020; Kommuri et al. 2025; Wang and Xu 2025).

Local delivery of statins directly into periodontal pockets or surgical sites has emerged as a promising therapeutic approach, minimizing systemic exposure while maximizing local bioavailability (Pradeep et al. 2015). A recent systematic review and network meta‐analysis evaluated adjunctive local therapies combined with NSPT in patients with type 2 diabetes mellitus and periodontitis, reporting improvements in probing pocket depth (PPD) and clinical attachment level (CAL) with statins (Lin et al. 2024). Moreover, their potential to enhance alveolar bone regeneration prior to dental implant placement and during simultaneous dental implant placement is currently being evaluated (Kommuri et al. 2025; Issa et al. 2024).

Although multiple clinical studies have demonstrated the benefits of locally delivered statins in periodontal therapy and pre‐implant bone regeneration (Ng et al. 2021; Kellesarian et al. 2017), findings remain inconclusive due to variability in statin type, concentration, delivery method, and clinical indications. A recent systematic review and meta‐analysis by Greethurst et al. reported beneficial adjunctive effects of locally delivered statins in periodontal therapy (Greethurst et al. 2024). However, this review primarily pooled clinical outcomes across statin types and treatment modalities, without differentiating between non‐surgical and surgical interventions, and did not incorporate evidence from pre‐implant bone regeneration procedures.

To date, few systematic reviews have addressed specific aspects of statin use (e.g., limited to NSPT or comparisons between local and systemic administration), and a comprehensive synthesis integrating non‐surgical (Step 2) and surgical (Step 3) periodontal therapies with pre‐implant bone regeneration procedures has not yet been performed. Accordingly, the present systematic review aimed to critically evaluate the clinical effectiveness and therapeutic potential of locally delivered statins, separately considering Step 2 non‐surgical therapy, Step 3 surgical therapy, and pre‐implant regenerative applications.

## 2 Materials and Methods

### 2.1 Protocol and Registration

**Figure 1:**
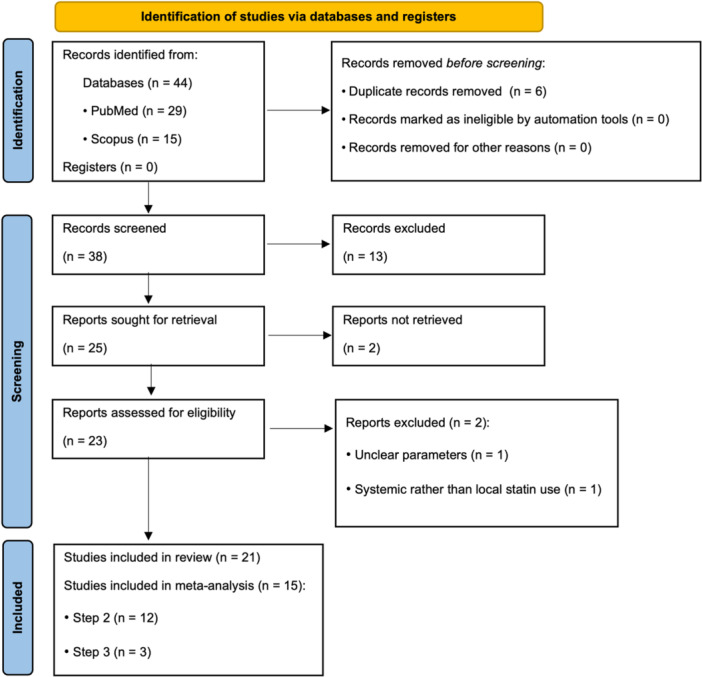
PRISMA flow diagram summarizing the study selection process (adapted from the PRISMA 2020 flow diagram template).

This systematic review was conducted in accordance with the Preferred Reporting Items for Systematic reviews and Meta‐Analyses (PRISMA) 2020 Guidelines (Page et al. 2021). The study selection process is presented in the PRISMA 2020 flow diagram (Figure 1), which was prepared using the official PRISMA 2020 template.

The review protocol was registered in the International Prospective Register of Systematic Reviews (PROSPERO; CRD420251105739) and is available at https://www.crd.york.ac.uk/PROSPERO/view/CRD420251105739.

### 2.2 Data Sources and Search Strategy

PubMed and Scopus electronic databases were searched until July 2025. The reference lists of eligible articles were also screened to identify additional relevant studies not retrieved through the electronic search strategy. The search strategy used in different databases is provided in Tables 1 and 2. Keywords were used individually and in combination to maximize search sensitivity. Boolean operators such as “AND” and “OR” were applied to combine search terms. Broad and umbrella terms (e.g., “periodontal disease,” “implant treatment”) were included in the search strategy to increase sensitivity. However, current terminology (“periodontitis,” “pre‐implant bone regeneration”) is used throughout the manuscript for consistency and accuracy.

**Table 1:** Search algorithm for PubMed.

Search order	Search string	Results
#1	“Hydroxymethylglutaryl‐CoA Reductase Inhibitors” [MeSH Terms] OR statin*[tiab]	7985
#2	“bone regeneration”[tiab] OR scaling[tiab] OR “root planing”[tiab]	126,461
#3	“periodontitis”[MeSH Terms] OR “periodontal disease”[tiab] OR “periodontal treatment”[tiab] OR “dental implant”[tiab] OR “implant treatment”[tiab]	32,564
#4	#1 AND #2 AND #3 AND (“clinical trial”[pt] OR “randomized controlled trial”[pt] OR clinical trial[tiab])	29

*Note:* Search details (including PubMed automatic term mapping) were checked, and the final search query was recorded. Results correspond to a PubMed search performed on July 10, 2025.

**Table 2:** Search algorithm for Scopus.

Search order	Corrected search string	Results
#1	TITLE‐ABS‐KEY (statin*)	221,644
#2	TITLE‐ABS‐KEY (“bone regeneration” OR “root planing” OR scaling)	1,751,107
#3	TITLE‐ABS‐KEY (periodontitis OR “periodontal treatment” OR “periodontal disease” OR “dental implant” OR “implant treatment”)	306,073
#4	#1 AND #2 AND #3 AND (LIMIT‐TO (EXACTKEYWORD, “Clinical Trial”))	15

*Note:* Results correspond to a Scopus search performed on July 10, 2025.

### 2.3 Inclusion and Exclusion Criteria

This systematic review addressed the question: “What is the effect of locally delivered statins on clinical outcomes when used as an adjunct to periodontal therapy and in pre‐implant bone regeneration procedures?”

The PICO model was applied to clearly structure the research question:

P (Population): Adults undergoing Step 2 subgingival instrumentation/NSPT, Step 3 periodontal therapy (periodontal surgery), Step 4 supportive periodontal care (SPC), or pre‐implant bone regeneration procedures.
I (Intervention): Local application of statins as an adjunct.
C (Comparison): Identical procedures with or without adjunctive statins.
O (Outcomes): Clinical periodontal parameters (PPD, CAL, bleeding on probing “BoP,” gingival index “GI,” plaque index “PI”) and radiographic bone outcomes, where applicable.

All clinical studies were screened, but only randomized controlled trials (RCTs) were included in the systematic review. Trials with extractable numerical data were included in the meta‐analysis.

Treatment modalities were classified according to the European Federation of Periodontology (EFP) S3 clinical practice guidelines (Herrera et al. 2022; Sanz et al. 2020), differentiating Step 2 (subgingival instrumentation/NSPT), Step 3 (periodontal surgery), and Step 4 (SPC).

Trials including patients in Step 4/SPC were analyzed separately as maintenance‐phase populations and were not pooled with active surgical (Step 3) trials; outcomes from Step 4/SPC studies were synthesized narratively.

### 2.4 Study Selection

Article selection was carried out in a multi‐step process to ensure methodological rigor. Duplicates were removed, and two independent reviewers (S. H. and N. M.) screened titles and abstracts for relevance. Full texts of potentially eligible articles were retrieved to confirm eligibility according to the predefined inclusion criteria. Any disagreements between reviewers were resolved through discussion with a third expert reviewer (F. A.).

### 2.5 Data Extraction and Study Quality Assessment

Data were extracted for study design, geographic location, year of publication, sample size, type of statin used, and clinical outcomes reported.

The risk of bias (RoB) in the included studies was assessed independently by two reviewers (S. H. and N. M.) using the Cochrane Risk of Bias 2 tool (RoB 2), the recommended framework for evaluating randomized trials (Higgins and Altman 2008; Higgins et al. 2011; Sterne et al. 2019).

RoB 2 evaluates five domains of potential bias: (1) bias arising from the randomization process, (2) bias due to deviations from intended interventions, (3) bias due to missing outcome data, (4) bias in measurement of the outcome, and (5) bias in selection of the reported result.

Each domain was judged as low risk, some concerns, or high risk.

For the purposes of summarizing the results in Table 3, a plus sign (+) indicates low risk, whereas a minus sign (−) represents either some concerns or high risk, which were grouped together because several studies lacked methodological detail sufficient to distinguish between these two categories. In addition, we recorded whether each study reported conflicts of interest, included as a separate column in Table 3.

**Table 3:** Overview of all assessments according to RoB.

References	Randomization	Deviations from intended intervention	Loss to follow‐up	Measurement	Selective reporting	Conflicts of interest (COI)
Garg and Pradeep (2017)	+	+	+	+	+	+
Gunjiganur Vemanaradhya et al. (2017)	+	+	+	+	+	+
Kumari et al. (2016)	−	+	+	+	+	+
Kumari et al. (2017)	+	+	−	+	+	+
Pankaj et al. (2018)	+	+	+	+	+	+
Pradeep and Thorat (2010)	−	+	−	+	+	+
Pradeep et al. (2012)	+	+	+	+	+	+
Pradeep, Kumari, et al. (2013)	+	+	−	+	+	+
Pradeep, Rao, et al. (2013)	+	+	−	+	+	+
Pradeep et al. (2015)	+	+	−	+	+	+
Pradeep, Garg, et al. (2016b)	+	+	+	+	+	+
Pradeep et al. (2017)	+	+	−	+	+	+
Rao et al. (2013)	+	+	−	+	+	+
Christiansen et al. (2023)	+	+	+	+	+	+
Killeen et al. (2022)	+	+	+	+	−	+
Issa et al. (2020)	+	+	+	+	+	+
Martande et al. (2016)	+	+	+	+	+	+
Pradeep, Garg, et al. (2016b)	+	+	+	+	+	+
Pradeep, Karvekar, et al. (2016)	+	+	+	+	+	+
Cruz et al. (2021)	+	+	+	+	+	+
Yaghobee et al. (2020)	+	+	−	+	+	+

*Note:* A plus sign (+) indicates a low risk of bias, while a minus sign (−) indicates either “some concerns” or high risk of bias according to the RoB 2 tool. These two categories were grouped together because insufficient methodological reporting often precluded reliable differentiation between some concerns and high risk.COI column: “+” indicates no conflict of interest declared (with disclosure statement provided); “−” indicates conflict of interest reported or no disclosure statement provided.

Two reviewers (S. H. and N. M.) performed all assessments independently, and any disagreements were resolved by discussion or consultation with a third expert reviewer (F. A.). A significant proportion of the included studies in this review were conducted by the same research group (Pradeep et al.), which may limit the generalizability of the findings (Pradeep et al. 2012, 2015, 2017; Pradeep, Kumari, et al. 2013; Pradeep, Rao, et al. 2013; Pradeep, Garg, et al. 2016a; Pradeep, Garg, et al. 2016b; Pradeep, Karvekar, et al. 2016; Pradeep and Thorat 2010). An overview of all RoB assessments is presented in Table 3.

Eligibility criteria and baseline thresholds were recorded verbatim when available (e.g., minimum PD/CAL/defect depth); otherwise, baseline descriptive values were summarized in the corresponding study characteristics tables (Tables 4, 5, 6).

**Table 4:** Characteristics of the controlled clinical studies on the use of statins in non‐surgical periodontal treatment.

References	Patients (*n*)	Male/Female	Age (years)	Smoking status	Follow‐up (months)	Loss to follow‐up	Treatment (test vs. control)	Agent, dose	Application (frequency, duration)	Adverse effects
Garg and Pradeep (2017)	90	N/A	N/A	No	3, 6, and 9	0	Test I: SRP + 1.2% RSV gel Test II: SRP + 1.2% ATV gel Control: SRP + placebo gel	Rosuvastatin (1.2%), Atorvastatin (1.2%)	BL and 6 months	None reported
Gunjiganur Vemanaradhya et al. (2017)	90	N/A	N/A	No	1.5	0	SRP + 1.2% SIM gel versus SRP	Simvastatin (1.2%)	BL	None reported
Kumari et al. (2016)	75	38/37	40–50	No	3, 6, and 9	15	SRP + 1.2% ATV gel versus SRP + placebo gel	Atorvastatin (1.2%)	BL	None reported
Kumari et al. (2017)	71	N/A	30–50	Yes (all)	3, 6, and 9	5	SRP + 1.2% ATV gel versus SRP + placebo gel	Atorvastatin (1.2%)	BL	None reported
Pankaj et al. (2018)	90	44/46	N/A	No	6 and 12	0	Test I: SRP + 1.2% RSV gel Test II: SRP + 1% MF gel Control: SRP + placebo gel	Rosuvastatin (1.2%), Metformin (1%)	BL	None reported
Pradeep and Thorat (2010)	64	33/31	25–45	No	1, 2, 4, and 6	4	SRP + 1.2% SIM gel versus SRP + placebo gel	Simvastatin (1.2%)	BL	None reported
Pradeep et al. (2012)	68	38/34	N/A	No	3 and 6	6	SRP + 1.2% SIM gel versus SRP + placebo gel	Simvastatin (1.2%)	BL	None reported
Pradeep, Kumari, et al. (2013)	67	35/32	30–50	No	3, 6, and 9	6	SRP + 1.2% ATV gel versus SRP + placebo gel	Atorvastatin (1.2%)	BL	None reported
Pradeep, Rao, et al. (2013)	38	20/18	30–50	No	3, 6, and 9	3	SRP + 1.2% SIM versus SRP + placebo gel	Simvastatin (1.2%)	BL	None reported
Pradeep et al. (2015)	70	33/37	25–55	No	1, 3, 4, and 6	5	SRP + 1.2% RSV gel versus SRP + placebo gel	Rosuvastatin (1.2%)	BL	None reported
Pradeep, Garg, et al. (2016a)	90	45/45	25–45	No	6 and 9	9	Test I: SRP + 1.2% RSV gel Test II: SRP + 1.2% ATV gel Control: SRP + placebo gel	Rosuvastatin (1.2%), Atorvastatin (1.2%)	BL and 6 months	None reported
Pradeep et al. (2017)	104	53/51	30–50	No	3, 6, and 9	14	Test I: SRP + 1.2% ATV gel Test II: SRP + 1% ALN Control: SRP + placebo gel	Atorvastatin (1.2%) Alendronate (1%)	BL	None reported
Rao et al. (2013)	40	40/0	30–50	Yes (all)	3, 6, and 9	5	SRP + 1.2% SIM gel versus SRP + placebo gel	Simvastatin (1.2%)	BL	None reported

*Note:* Baseline (BL) indicates the start of treatment or assessment.

Abbreviations: ALN, alendronate; ATV, atorvastatin; MF, metformin; N/A, not available; RSV, rosuvastatin; SIM, simvastatin; SRP, scaling and root planing.

**Table 5:** Characteristics of the controlled clinical studies on the use of statins in surgical periodontal treatment.

References	Patients (*n*)	Male/Female	Age (years)	Smoking status	Follow‐up (months)	Loss to follow‐up	Treatment (test vs. control)	Drug, dose	Application: Defect type; frequency; duration	Adverse effects	Findings
Step 4 (supportive periodontal care/maintenance‐phase therapy)											
Christiansen et al. (2023)	50	N/A	N/A	Smokers: 30% (test) versus 4% (control)	12	2	PMT + PR/SRP + 1.2% SIM gel versus PMT + PR/SRP + placebo gel	Simvastatin (1.2%)	BL	None reported	•Both groups showed improvements in BoP*, with greater improvement in the test group (no significant differences between groups).
											•Both groups showed improvements in CAL compared to baseline*, and the test group showed greater improvement compared to the control group*.
Killeen et al. (2022)	50	30/20	N/A	Smokers: 29.6% (test) versus 4.4% (control)	12	2	PR/RP + 2.2 mg SIM gel (0.15 ml MCL) versus PR/RP + placebo gel	Simvastatin (2.2 mg/0.15 ml MCL), EDTA (concentration not specified)	BL	Reported adverse reactions included transient temperature sensitivity (18.7%), pain (12.5%), and swelling (< 0.1%).	•Addition of SIM gel showed improvements in CAL, PPD, and BoP compared to the control group*.
Step 3 (active surgical periodontal therapy)											
Issa et al. (2020)	40	12/28	30–50	No	6 and 9	0	Test I: 1.2% SIM gel + OM; Test II: 1.2% SIM gel + PBM; Test III: EDTA + 1.2% SIM gel + OM; Test IV: EDTA + 1.2% SIM gel + PBM	Simvastatin (1.2%), EDTA (24%)	Angular bone defect BL	None reported	•All groups showed improvements in PPD and CAL at 6 and 9 months compared to baseline*.
											•No statistically significant differences in PPD and CAL were observed between Group III and Group IV (EDTA added).
											•Both Group III and Group IV showed greater improvement in PPD and CAL compared to Group I and Group II*.
											•Group IV showed increased bone defect fill compared to the other groups*.
											•Group II showed greater bone defect fill compared to Group I and Group III.
											•Bone density increased in all four groups* with no statistically significant differences between the groups.
Martande et al. (2016)	96	48/48	30–50	No	9	6	Test I: OFD + PRF Test II: OFD + PRF + 1.2% ATV gel Control: OFD	Atorvastatin (1.2%)	Angular bone defect BL	None reported	•All groups showed improvements in PPD, CAL, and bone defect fill*.
											•The addition of 1.2% ATV gel did not result in improvements compared to treatment with PRF alone, except in terms of bone defect fill*.
Pradeep, Garg, et al. (2016b)	90	45/45	25–45	No	9	0	Test I: OFD + PRF Test II: OFD + PRF + 1.2% RSV gel Control: OFD	Rosuvastatin (1.2%)	Angular bone defect BL	None reported	•The group treated with PRF showed improvements in CAL, PPD, and a reduction in the depth of IBDs compared to the control group*.
											•The addition of 1.2% RSV gel to PRF resulted in improvements in CAL, PPD, and a reduced depth of IBDs compared to the PRF group*.
Pradeep, Karvekar, et al. (2016)	110	60/50	N/A	No	9	5	Test I: PRF + HA with OFD Test II: 1.2% RSV gel + PRF + HA with OFD Control: OFD + placebo gel	Rosuvastatin (1.2%), platelet‐rich fibrin, porous hydroxyapatite bone graft	Furcation involvement Grade II in the mandible BL	None reported	•1.2% RSV gel combined with autologous PRF and porous HA bone graft resulted in improvements in clinical and radiographic parameters (PI, BoP, PPD, CAL)* compared to OFD alone, in the treatment of furcation‐involved teeth.
											•Improvements in PPD and CAL were also observed compared to OFD + HA + PRF alone*.
											•The results suggest that RSV, PRF, and HA may have a synergistic effect.

Abbreviations: ATV, atorvastatin; BL, baseline; BoP, bleeding on probing; CAL, clinical attachment level; EDTA, ethylenediaminetetraacetic acid; HA, hydroxyapatite; IBDs, intrabony defects; MCL, methylcellulose; N/A, not available; OFD, open flap debridement; OM, occlusive membranes; PBM, perforated barrier membrane; PI, plaque index; PMT, periodontal maintenance therapy; PPD, probing pocket depth; PR, papilla reflection; PRF, platelet‐rich fibrin; RSV, rosuvastatin; SIM, simvastatin; SRP, scaling and root planing.

* Statistically significant (*p* < 0.05).

**Table 6:** Characteristics of the controlled clinical studies on the use of statins in pre‐implant bone regeneration procedures/implant site development.

References	Patients (*n*)	Male/Female	Age (years)	Smoking status	Follow‐up (months)	Loss to follow‐up	Treatment (test vs. control)	Agent, dose	Applications: Defect type frequency duration	Adverse effects	Findings
Cruz et al. (2021)	33	N/A	N/A	No	3	7	Socket filled with 1.2% SIM gel and covered with PPPM versus a socket filled with placebo gel and covered with PPPM	Simvastatin (1.2%)	Extraction socket BL	None reported	•At 3 months post‐extraction, a statistically significant difference in bone width was observed at all three measured levels compared to the control group. •The test group showed a statistically significant difference in alveolar depth compared to the control group.
											•No differences were found between the groups in soft tissue healing, perceived pain, or the amount of analgesics consumed.
Yaghobee et al. (2020)	12	8/4	53–67	≤ 10 cigarettes per day allowed	9	1	BBM + SIM versus BBM	Simvastatin (1.6 g/100 mL 70% ethanol). 8.33 mg Simvastatin/0.5 mL BBM.	Sinus augmentation BL	None reported	•After 9 months, both groups showed approximately 20% increased bone growth, with no statistically significant differences. •A slightly greater amount of mineralized tissue was observed in the test group.

Abbreviations: BBM, bovine bone material; BL, baseline; N/A, not available; PPPM, polypropylene membranes; SIM, simvastatin.

### 2.6 Meta‐Analysis

The selection of follow‐up time points for quantitative synthesis was defined a priori as part of the data synthesis plan. Meta‐analysis was conducted only at follow‐up intervals consistently reported in at least three RCTs and considered biologically and clinically relevant for periodontal and bone healing.

For NSPT (Step 2), 6‐month follow‐up data were selected, as this was the most frequently reported time point across eligible trials and reflects a clinically meaningful period for evaluating changes in PPD and CAL.

For surgical periodontal therapy (Step 3), 9‐month follow‐up data were selected, as this was the only time point consistently reported across at least three studies and corresponds to a biologically relevant phase of periodontal wound healing and bone maturation.

Step 4/SPC trials represented maintenance‐phase populations and were excluded a priori from quantitative pooling, being synthesized narratively.

Meta‐analysis was performed only when studies reported extractable numerical data, including sample size, mean changes from baseline, and corresponding standard deviations for PPD and CAL at a shared follow‐up interval. Although all non‐surgical and surgical studies are summarized in Tables 4 and 5, only those providing complete numerical datasets were eligible for pooling.

**Figure 2:**
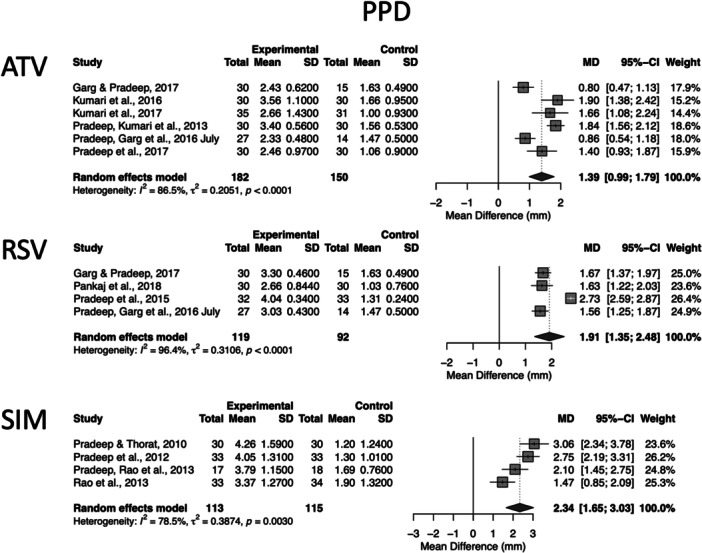
Six‐month changes in PPD following Step 2 periodontal therapy with adjunctive locally delivered statins. Forest plots show pooled mean differences (MDs) and 95% confidence intervals comparing statin groups (ATV, RSV, SIM, analyzed separately) with scaling and root planing (SRP) alone using a random‐effects model. Heterogeneity statistics (*I*², *τ*², and Cochran's *Q*) are reported for each subgroup. In multi‐arm trials with a shared placebo control, the control group sample size was evenly split across comparisons to avoid double‐counting. Positive MD values indicate greater improvement in the statin group (i.e., larger PPD reduction).

**Figure 3:**
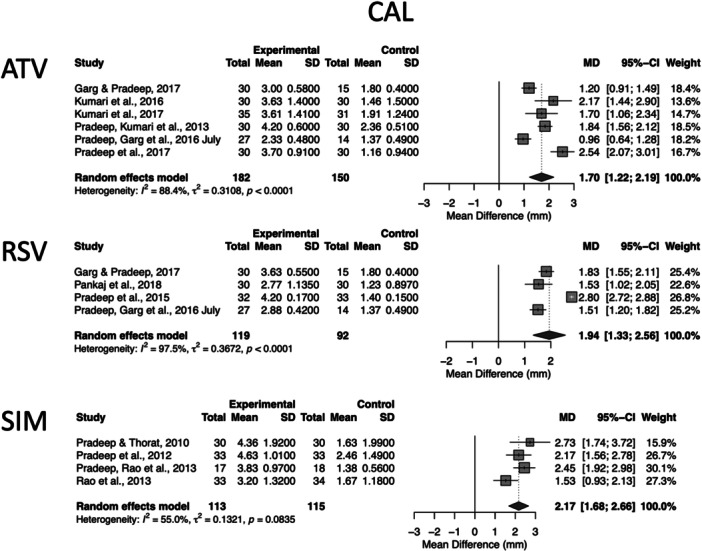
Six‐month changes in CAL following Step 2 periodontal therapy with adjunctive locally delivered statins. Forest plots show MDs and 95% confidence intervals comparing ATV, RSV, and SIM (analyzed separately using random‐effects models) with scaling and root planing (SRP) alone. Heterogeneity statistics (*I*², *τ*², and Cochran's *Q*) are reported for each subgroup. Shared placebo control groups in multi‐arm trials were handled by splitting the control sample size evenly across comparisons. Positive MD values indicate greater improvement in the statin group (i.e., larger CAL gain).

For non‐surgical therapy (Step 2), 14 extractable datasets derived from 12 unique RCTs provided complete 6‐month change data (PPD and CAL) and were included in the pooled analyses. These consisted of six atorvastatin (ATV) studies, including Garg and Pradeep (2017), Kumari et al. (2016, 2017), Pradeep, Kumari, et al. (2013), Pradeep, Garg, et al. (2016a, July), and Pradeep et al. (2017); four rosuvastatin (RSV) studies, including Garg and Pradeep (2017), Pankaj et al. (2018), Pradeep et al. (2015), and Pradeep, Garg, et al. (2016a); and four simvastatin (SIM) studies, including Pradeep and Thorat (2010), Pradeep et al. (2012), Pradeep, Rao, et al. (2013b), and Rao et al. (2013). These studies provided the full numerical data required for quantitative synthesis and enabled three separate statin‐specific pooled analyses, presented in Figures 2 and 3.

Because two of the included RCTs, Pradeep, Garg, et al. (2016a) and Garg and Pradeep (2017), reported two independent statin intervention arms (ATV and RSV) compared with a shared placebo control group, each study contributed two extractable datasets. Therefore, although the meta‐analysis included 14 datasets, these were derived from 12 distinct studies. In accordance with Cochrane recommendations for multi‐arm trials, the shared control groups in these studies were evenly divided across the relevant comparisons, while means and standard deviations were kept unchanged, ensuring correct weighting and avoiding unit‐of‐analysis errors. This sample‐size splitting approach does not reflect the correlation between comparisons within multi‐arm trials; therefore, results should be interpreted as approximate.

One non‐surgical study (Gunjiganur Vemanaradhya et al. 2017) was included in the qualitative synthesis but excluded from the 6‐month meta‐analysis because it reported only a 45‐day follow‐up and did not provide numerical 6‐month PPD or CAL data (Gunjiganur Vemanaradhya et al. 2017).

**Figure 4:**
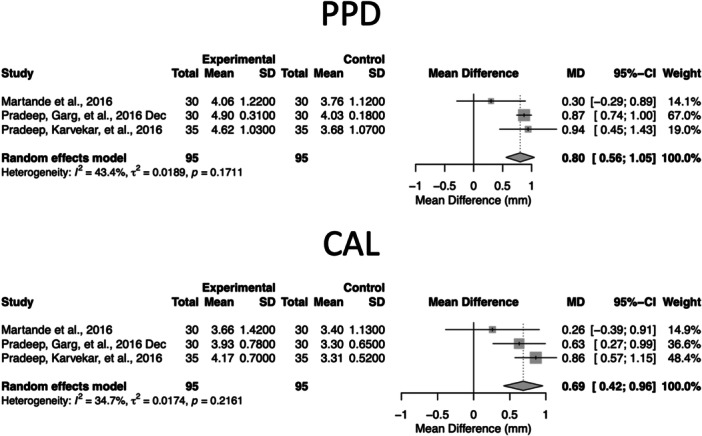
Forest plots of 9‐month changes in PPD and CAL following Step 3 periodontal surgery with adjunctive locally delivered statins. Forest plots show MDs and 95% confidence intervals for trials reporting extractable 9‐month data comparing surgery with adjunctive statins versus control procedures. A random‐effects model was applied; heterogeneity measures (*I*², *τ*², and Cochran's *Q*) are displayed. Positive MD values indicate greater improvement in the statin group (i.e., larger PPD reduction and CAL gain).

For surgical therapy, only three trials reported complete 9‐month PPD and CAL datasets, including Martande et al. (2016), Pradeep, Garg, et al. (2016b), and Pradeep, Karvekar, et al. (2016). These were therefore included in the pooled surgical meta‐analysis. The resulting 9‐month effect estimates are presented in Figure 4.

Other follow‐up intervals did not provide sufficient numerical compatibility for pooling and were narratively synthesized. Pre‐implant bone regeneration studies also lacked compatible numerical data and were summarized narratively.

K. Y. manually extracted all numerical values used for the meta‐analysis from the tables presented in the full texts of the included articles. The extracted data were independently cross‐checked by L. O., with discrepancies resolved through consensus or consultation with F. A. Data in Tables 4 and 5 were used only as descriptive summaries and not as quantitative sources. Meta‐analysis was conducted in R (version 4.5.0) using the meta package with a random‐effects model (REML). Heterogeneity was assessed via *I*², *τ*², and Cochran's *Q*.

Given the substantial heterogeneity anticipated a priori and observed in the analyses, particularly in the non‐surgical comparisons where *I*² values often exceeded 80%–90%, random‐effects models were primarily used to summarize the overall direction of effects rather than to estimate a single common effect. This marked heterogeneity was likely driven by differences in statin type and concentration, dosing protocols, baseline periodontal severity, defect morphology, adjunctive materials, and follow‐up duration. These comparisons remain indirect.

Publication bias assessment using funnel plot inspection and Egger's regression test was planned when ≥ 10 comparisons were available; however, none of the statin‐specific meta‐analysis included ≥ 10 comparisons, so these analyses were not performed.

Mean differences (MDs) were used as the primary effect measure because PPD and CAL are clinically interpretable in millimeters. Although baseline periodontal parameters differed among trials, standardized MDs were not calculated, as MDs provide more direct clinical meaning. This choice should be considered when interpreting pooled effects.

For meta‐analysis, we extracted mean changes from baseline (and corresponding standard deviations) in PPD and CAL at the prespecified follow‐up time points. Accordingly, pooled MDs represent differences in change from baseline between test and control groups.

## 3 Results

### 3.1 Study Selection and Literature Search

Figure 1 presents the PRISMA flow diagram summarizing the identification, screening, eligibility assessment, and final inclusion of studies. It outlines the number of records retrieved, screened, and excluded, and the reasons for full‐text exclusion, providing a transparent overview of the study selection process.

A total of 44 records were retrieved (29 from PubMed and 15 from Scopus). After removing 6 duplicates, 38 records remained for screening. Abstract screening resulted in 25 articles, of which 2 could not be retrieved in full text. Of the remaining 23 articles assessed for eligibility, 2 were excluded after full‐text review: Rahman et al. due to unclear parameters (Rahman et al. 2020), and Fajardo et al. for focusing on systemic rather than local treatment (Fajardo et al. 2010). A total of 21 studies were included in this systematic review. Fifteen studies contributed numerical data to the quantitative syntheses. Specifically, 12 Step 2 trials contributed to the 6‐month statin‐specific meta‐analysis, and 3 Step 3 trials contributed to the 9‐month surgical meta‐analysis, while the remaining studies were included only in the qualitative review.

### 3.2 RoB Assessment

Overall, more than two‐thirds of the included RCTs were judged as having “some concerns” in at least one domain of the RoB 2 assessment. The most frequently observed issues were incomplete reporting of the randomization process and allocation concealment, limited details on blinding of participants or outcome assessors, and insufficient methodological information regarding missing outcome data or selective reporting of results. In most cases, these risk‐of‐bias assessments reflected incomplete reporting rather than clear methodological issues; however, they may reduce confidence in the precision and reproducibility of the observed treatment effects.

### 3.3 Study Population

Across the included studies, diagnostic definitions and baseline disease severity varied considerably. Most non‐surgical and surgical trials enrolled patients described as having chronic periodontitis or moderate‐to‐severe periodontitis according to contemporary criteria at the time of publication, typically defined by PPD thresholds of ≥ 5 or ≥ 6 mm and the presence of intrabony or furcation defects. Formal staging and grading according to the 2018 classification were not applied in the original trials (Tonetti et al. 2018; Papapanou et al. 2018). Detailed information on diagnostic criteria, defect types, and baseline disease severity across studies is provided in Table S1.

#### 3.3.1 Step 2 Periodontal Therapy (NSPT)

Thirteen studies evaluating Step 2 periodontal therapy (NSPT) in patients with periodontitis included 38–104 patients. Four did not report age (Pradeep et al. 2012; Garg and Pradeep 2017; Pankaj et al. 2018; Gunjiganur Vemanaradhya et al. 2017), whereas others included patients aged 25–55 years (Pradeep et al. 2015, 2017; Pradeep, Kumari, et al. 2013; Pradeep, Rao, et al. 2013; Pradeep, Garg, et al. 2016a; Pradeep and Thorat 2010; Kumari et al. 2016, 2017; Rao et al. 2013). Gender was not reported in three studies (Garg and Pradeep 2017; Kumari et al. 2017; Gunjiganur Vemanaradhya et al. 2017), and one included only men (Rao et al. 2013). Nine studies had equal gender distribution (Pradeep et al. 2012, 2015, 2017; Pradeep, Kumari, et al. 2013; Pradeep, Rao, et al. 2013; Pradeep, Garg, et al. 2016a; Pradeep and Thorat 2010; Kumari et al. 2016; Pankaj et al. 2018). Two studies involved only smokers (Kumari et al. 2017; Rao et al. 2013), while smoking was an exclusion criterion in the remaining 11 studies (Pradeep et al. 2012, 2015, 2017; Pradeep, Kumari, et al. 2013; Pradeep, Rao, et al. 2013; Pradeep, Garg, et al. 2016a; Pradeep and Thorat 2010; Garg and Pradeep 2017; Kumari et al. 2016; Pankaj et al. 2018; Gunjiganur Vemanaradhya et al. 2017).

In these Step 2 studies, statin therapy was mainly applied to sites with deep periodontal pockets and intrabony defects, with baseline mean PPD values generally exceeding 6 mm or with inclusion criteria specifying pockets ≥ 5–6 mm. Thus, the non‐surgical evidence largely reflects treatment effects in moderate‐to‐severe periodontal disease rather than shallow or early‐stage disease.

#### 3.3.2 Step 3 Periodontal Therapy (Periodontal Surgery)

Six studies on the surgical treatment of periodontitis included 40–110 patients. Three did not report age (Pradeep, Karvekar, et al. 2016; Christiansen et al. 2023; Killeen et al. 2022); two included patients aged 30–50 (Martande et al. 2016; Issa et al. 2020), and one reported an age range of 25–45 years (Pradeep, Garg, et al. 2016b). Gender distribution was not reported in one study (Christiansen et al. 2023), equally distributed in two (Pradeep, Garg, et al. 2016b; Martande et al. 2016), and varied across the remaining three studies (Pradeep, Karvekar, et al. 2016; Killeen et al. 2022; Issa et al. 2020).

Smoking was excluded in four studies (Pradeep, Garg, et al. 2016b; Pradeep, Karvekar, et al. 2016; Martande et al. 2016; Issa et al. 2020), but included in two (Christiansen et al. 2023; Killeen et al. 2022).

Step 3 studies primarily targeted advanced periodontal defects, including angular intrabony defects and Class II furcation involvement, and were often performed within regenerative surgical protocols involving adjunctive biomaterials. Of the six surgical‐periodontal studies, two trials (Christiansen et al. 2023; Killeen et al. 2022) enrolled periodontal maintenance patients (Step 4/SPC) rather than active disease populations (Christiansen et al. 2023; Killeen et al. 2022).

#### 3.3.3 Pre‐Implant Bone Regeneration Procedures

Two studies investigated the use of statins as adjuncts in pre‐implant bone regeneration procedures, involving 24 to 33 patients (Table 6). Cruz et al. (2021) did not provide information on age or gender. In contrast, Yaghobee et al. (2020) reported that male participants outnumbered females two to one, with an age range of 53 to 67 years. Smoking was excluded in the Cruz study, while the Yaghobee study allowed up to 10 cigarettes per day.

These studies addressed distinct clinical scenarios, socket preservation following tooth extraction and sinus augmentation.

### 3.4 Treatment Methods

Statins were evaluated as adjuncts to non‐surgical periodontal treatment in 13 studies (Pradeep et al. 2012, 2015, 2017; Pradeep, Kumari, et al. 2013; Pradeep, Rao, et al. 2013; Pradeep, Garg, et al. 2016a; Pradeep and Thorat 2010; Garg and Pradeep 2017; Kumari et al. 2016, 2017; Pankaj et al. 2018; Rao et al. 2013; Gunjiganur Vemanaradhya et al. 2017), in 6 studies on surgical treatment (Pradeep, Garg, et al. 2016b; Pradeep, Karvekar, et al. 2016; Martande et al. 2016; Christiansen et al. 2023; Killeen et al. 2022; Issa et al. 2020), and in 2 studies prior to implant placement (Cruz et al. 2021; Yaghobee et al. 2020).

Table 7 summarizes the substances, application methods, frequencies, and outcomes. Three statins were used: ATV, RSV, and SIM. All included studies used locally delivered statin formulations; most periodontitis treatment trials (Step 2 and Step 3, including maintenance/SPC populations) applied statins as 1.2% methylcellulose gels delivered subgingivally, whereas the two pre‐implant bone regeneration trials applied statin formulations directly into extraction sockets or within sinus graft materials. Most studies applied the gel at baseline (Pradeep et al. 2012, 2015, 2017; Pradeep, Kumari, et al. 2013; Pradeep, Rao, et al. 2013; Pradeep, Garg, et al. 2016b; Pradeep, Karvekar, et al. 2016; Pradeep and Thorat 2010; Garg and Pradeep 2017; Kumari et al. 2016, 2017; Pankaj et al. 2018; Rao et al. 2013; Gunjiganur Vemanaradhya et al. 2017; Martande et al. 2016; Christiansen et al. 2023; Killeen et al. 2022; Issa et al. 2020; Cruz et al. 2021; Yaghobee et al. 2020), while only two reported a second application at 6 months (Pradeep, Garg, et al. 2016a; Garg and Pradeep 2017).

**Table 7:** Summary of findings from 21 controlled trials on local statin delivery as adjunct to treatment.

Summary of findings from 21 controlled studies on statin treatment
**Application method**
▪Three different statins were used across the included studies: Simvastatin (SIM), Rosuvastatin (RSV), and Atorvastatin (ATV).
▪Frequency of use: 19 studies applied the statin gel only at baseline (BL), while 2 studies reapplied it at 6 months.
▪Duration of follow‐up: 1 study reported a 1.5‐month follow‐up period; the most common follow‐up was 9 months, and 2 studies followed participants for 12 months.
▪Application method: All 21 studies used local statin delivery; most periodontal studies applied methylcellulose‐based gels subgingivally, whereas the two implant‐related trials applied statins directly into extraction sockets or within sinus graft materials.
▪Reported side effects: 20 studies reported no adverse effects; 1 study reported transient temperature sensitivity, pain, and swelling.
**Results of Step 2 periodontal therapy (subgingival instrumentation/NSPT; 13 studies)**
▪12 studies reported statistically significant reductions in PPD.
▪12 studies reported statistically significant gains in CAL.
▪9 studies reported statistically significant reductions in BoP.
▪4 studies reported statistically significant reductions in GI.
▪2 studies reported statistically significant reductions in PI.
**Results of Step 3 periodontal therapy (periodontal surgery; 6 studies)**
▪4 studies reported statistically significant reductions in PPD.
▪2 studies reported statistically significant reductions in BoP.
▪2 studies reported statistically significant reductions in the depth of angular bone defects.
▪1 study reported a statistically significant reduction in PI.
▪1 study reported that the combination of root surface conditioning with EDTA + SIM gel resulted in statistically significant improvements in both CAL and PPD compared to SIM gel alone.
▪1 study reported that the combination of EDTA + SIM gel + PBM resulted in a statistically significant reduction in the depth of angular bone defects compared to the absence of EDTA and use of a different membrane (OM).
▪1 study reported a potential synergistic effect of PRF + HA + RSV, demonstrating statistically significant improvements in CAL and PPD compared to PRF + HA alone.
**Results of pre‐implant bone regeneration procedures (2 studies)**
▪1 study reported statistically significant increases in bone width and reductions in alveolar depth at 3 months.
▪1 study reported comparable bone fill at 9 months when SIM was combined with BBM.

Abbreviations: ATV, atorvastatin; BBM, bovine bone mineral; BL, baseline; BoP, bleeding on probing; CAL, clinical attachment level; EDTA, ethylenediaminetetraacetic acid; GI, gingival index; HA, hydroxyapatite; OM, occlusive membrane; PBM, perforated barrier membrane; PI, plaque index; PPD, probing pocket depth; PRF, platelet‐rich fibrin; RSV, rosuvastatin; SIM, simvastatin.

Twenty studies reported no adverse effects (Pradeep et al. 2012, 2015, 2017; Pradeep, Kumari, et al. 2013; Pradeep, Rao, et al. 2013; Pradeep, Garg, et al. 2016a, 2016b; Pradeep, Karvekar, et al. 2016; Pradeep and Thorat 2010; Garg and Pradeep 2017; Kumari et al. 2016, 2017; Pankaj et al. 2018; Rao et al. 2013; Gunjiganur Vemanaradhya et al. 2017; Martande et al. 2016; Christiansen et al. 2023; Issa et al. 2020; Cruz et al. 2021; Yaghobee et al. 2020), whereas one study reported transient adverse reactions (temperature sensitivity, pain, and swelling) (Killeen et al. 2022).

### 3.5 Description of Treatment Protocols

#### 3.5.1 Step 2 Periodontal Therapy (Subgingival Instrumentation/NSPT)

In the studies listed in Table 4, non‐surgical periodontal treatment involved the adjunctive use of ATV, RSV, or SIM in combination with scaling and root planing (SRP), a procedure also referred to as instrumentation or debridement.

#### 3.5.2 Step 3 Periodontal Surgery

In the studies on surgical periodontal treatment presented in Table 5, statin gels containing ATV, RSV, or SIM were commonly used. However, the adjunctive techniques and materials varied between studies.

Papilla reflection, a minimally invasive surgical method, was employed in two studies to enhance visibility and access for SRP (Christiansen et al. 2023; Killeen et al. 2022). In two studies, the root surface was conditioned with ethylenediaminetetraacetic acid (EDTA) before applying the statin gel (Killeen et al. 2022; Issa et al. 2020). In one of these, the gel was applied in angular bone defects, then covered with either an occlusive membrane (OM) or perforated barrier membrane (PBM) to support guided tissue regeneration (Issa et al. 2020). Platelet‐rich fibrin (PRF) was combined with statins in three studies to enhance treatment outcomes (Pradeep, Garg, et al. 2016b; Pradeep, Karvekar, et al. 2016; Martande et al. 2016). Among these, only one study investigated degree II furcation involvement in the mandible, employing statins, PRF, and a porous hydroxyapatite (HA) bone graft (Pradeep, Karvekar, et al. 2016).

#### 3.5.3 Pre‐Implant Bone Regeneration Procedures

In the two studies evaluating statins as adjuncts in pre‐implant bone regeneration procedures presented in Table 6, both used SIM (Cruz et al. 2021; Yaghobee et al. 2020). In the first study, SIM gel was applied directly into the extraction socket at the time of extraction and subsequently covered with a polypropylene membrane (PPPM) (Cruz et al. 2021). In the second study, the gel was combined with bovine bone material and applied following sinus augmentation (Yaghobee et al. 2020).

### 3.6 Therapeutic Effect

#### 3.6.1 Step 2 Periodontal Therapy (NSPT)

Across the 13 Step 2 trials, the adjunctive use of locally delivered statins resulted in consistently superior clinical outcomes compared with SRP alone.

Table 8 provides a visual summary of the direction of effects across PPD, CAL, BoP, GI, and PI, rather than presenting detailed numerical data from each individual study. Overall, most trials demonstrated clearly greater PPD reduction and CAL gain in statin‐treated sites, irrespective of whether ATV, RSV, or SIM was used (Pradeep et al. 2012, 2015, 2017; Pradeep, Kumari, et al. 2013; Pradeep, Rao, et al. 2013; Pradeep, Garg, et al. 2016a; Pradeep and Thorat 2010; Garg and Pradeep 2017; Kumari et al. 2016, 2017; Pankaj et al. 2018; Rao et al. 2013; Gunjiganur Vemanaradhya et al. 2017).

**Table 8:** Overview of clinical findings from non‐surgical periodontal treatment with adjunctive statins, compared to control groups.

References	Pocket depth	Clinical attachment level	Bleeding on probing	Gingival index	Plaque index
Garg and Pradeep (2017)	↑	↑	↑	N/A	=
Gunjiganur Vemanaradhya et al. (2017)	=	=	↑	↑	↑
Kumari et al. (2016)	↑	↑	↑	N/A	=
Kumari et al. (2017)	↑	↑	↑	N/A	=
Pankaj et al. (2018)	↑	↑	↑	N/A	=
Pradeep and Thorat (2010)	↑	↑	N/A	=	=
Pradeep et al. (2012)	↑	↑	N/A	↑	=
Pradeep, Kumari, et al. (2013)	↑	↑	↑	N/A	=
Pradeep, Rao, et al. (2013)	↑	↑	↑	N/A	=
Pradeep et al. (2015)	↑	↑	N/A	↑	N/A
Pradeep, Garg, et al. (2016a)	↑	↑	↑	N/A	↑
Pradeep et al. (2017)	↑	↑	↑	N/A	=
Rao et al. (2013)	↑	↑	N/A	↑	=

*Note:* (=), No statistically significant differences; (↑), Statistically significant difference (*p* < 0.05).

Abbreviation: N/A, not available.

BoP also improved in nine trials (Pradeep, Garg, et al. 2016a; Pradeep et al. 2017; Pradeep, Kumari, et al. 2013; Pradeep, Rao, et al. 2013; Garg and Pradeep 2017; Kumari et al. 2016, 2017; Pankaj et al. 2018; Gunjiganur Vemanaradhya et al. 2017), while four studies reported significant improvements in GI (Pradeep et al. 2012, 2015; Rao et al. 2013; Gunjiganur Vemanaradhya et al. 2017) and two reported reductions in PI (Pradeep, Garg, et al. 2016a; Gunjiganur Vemanaradhya et al. 2017).

Although these secondary measures were less consistent, their overall direction of effect was favorable for statin‐treated groups.

Taken together, the findings in Table 8 show a coherent pattern: locally delivered statins used as adjuncts to SRP provide meaningful improvements, most notably in PPD, CAL, with generally favorable trends in BoP, supporting their clinical application in NSPT (Pradeep et al. 2012, 2015, 2017; Pradeep, Kumari, et al. 2013; Pradeep, Rao, et al. 2013; Pradeep, Garg, et al. 2016a; Pradeep and Thorat 2010; Garg and Pradeep 2017; Kumari et al. 2016, 2017; Pankaj et al. 2018; Rao et al. 2013; Gunjiganur Vemanaradhya et al. 2017).

#### 3.6.2 Step 3 Periodontal Surgery

Across the six Step 3 trials evaluating statins as adjuncts to surgical periodontal therapy, Table 5 provides a detailed comparison of clinical and radiographic outcomes, including PPD, CAL, BoP, and radiographic defect fill.

Overall, statin‐treated groups consistently demonstrated superior outcomes compared with their respective controls, although the magnitude and nature of improvement varied depending on the surgical technique and adjunctive biomaterials used.

Most surgical trials reported greater CAL gain at statin‐treated sites; however, between‐group differences were not consistently statistically significant, particularly in the exploratory mini‐flap study by Christiansen et al. as summarized in Table 5 (Pradeep, Garg, et al. 2016b; Pradeep, Karvekar, et al. 2016; Martande et al. 2016; Christiansen et al. 2023; Killeen et al. 2022; Issa et al. 2020).

Radiographic outcomes were also generally improved, particularly when statins were combined with regenerative materials such as PRF, HA, EDTA, or barrier membranes. Notably, EDTA conditioning and PBM combined with SIM resulted in the greatest improvements, suggesting synergy with regenerative microenvironments.

Collectively, the available evidence suggests that statins may be beneficial adjuncts in surgical periodontal therapy, with the clearest gains observed when integrated into regenerative protocols.

Across the included trials, local statin administration as an adjunct to Step 2 (non‐surgical) and Step 3 (surgical) periodontal therapy was consistently associated with favorable radiographic outcomes, including reductions in infrabony defect depth and increases in alveolar bone density (Pradeep et al. 2013; Pradeep et al. 2015; Pradeep et al. 2016a; Pradeep et al. 2016b; Pradeep et al. 2017; Kumari et al. 2016; Kumari et al. 2017; Pankaj et al. 2018; Pradeep and Thorat 2010; Pradeep et al. 2012; Rao et al. 2013; Martande et al. 2016; Garg and Pradeep 2017; Pradeep, Karvekar et al. 2016). Comparisons between statins revealed some variability: RSV was associated with greater radiographic improvement compared to ATV in certain Step 2 studies (Pradeep et al. 2016a; Garg and Pradeep 2017). In other step 2 studies, RSV showed greater radiographic decrease in intrabony defect (Pradeep et al. 2015) and improved bone fill (Pankaj et al. 2018). Similarily, adjunctive use of SIM in step 2 periodontal therapy showed greater radiographic decrease in intrabony defect (Pradeep and Thorat 2010) and greater bone fill (Rao et al. 2013). Certain trials also evaluated statins against other adjunctive agents: RSV versus metformin showed no significant difference in radiographic outcomes (Pankaj et al. 2018), and ATV versus alendronate (ALN) showed greater defect depth reduction for ALN compared to ATV (Pradeep et al. 2017). In step 3 studies, RSV combined with PRF resulted in greater depth reductions of intrabony defects (Pradeep, Garg et al. 2016b), while ATV combined with PRF showed a similar percentage radiographic defect depth reduction, compared with PRF alone (Martande et al. 2016). Importantly, these benefits were observed not only in systemically healthy patients but also in smokers and well‐controlled type 2 diabetes patients (Kumari et al. 2016; Kumari et al. 2017; Rao et al. 2013), indicating that statin therapy may enhance bone regeneration across diverse clinical populations. Overall, the radiographic improvements observed support the clinical relevance of statins as adjunctive therapy in Step 2 and Step 3 periodontal treatment, contributing to more effective infrabony defect resolution.

#### 3.6.3 Pre‐Implant Bone Regeneration Procedures

Two clinical studies evaluated the use of locally delivered SIM as an adjunct to regenerative procedures performed prior to dental implant placement, as summarized in Table 6.

Findings were heterogeneous: one study (Cruz et al. 2021) showed enhanced early socket healing, significantly smaller horizontal ridge width loss, and reduced vertical dimensional change (alveolar depth) at 3 months, whereas the sinus augmentation study (Yaghobee et al. 2020) reported no significant differences between the statin and control groups at early or later follow‐up.

By 9 months, both groups in the sinus augmentation study demonstrated similar graft maturation, suggesting that any potential statin effect may be limited or overshadowed by the regenerative properties of the graft material.

Overall, evidence for statins in pre‐implant bone regeneration procedures remains limited and inconsistent, and the available data are insufficient to determine whether local statins provide a consistent or clinically meaningful benefit in pre‐implant bone regeneration procedures.

### 3.7 Meta‐Analysis

#### 3.7.1 Non‐Surgical Therapy (6‐Month Meta‐Analysis)

Figure 2 presents the forest plot for PPD, and Figure 3 presents the forest plot for CAL, each displaying pooled effects for ATV, RSV, and SIM. These two figures separately visualize the effect sizes, confidence intervals, study weights, and heterogeneity estimates for each clinical outcome, allowing a clearer outcome‐specific assessment of treatment effects and between‐study variability. The pooled datasets for Figures 2 and 3 were derived from six ATV studies (Pradeep, Kumari, et al. 2013a; Pradeep, Garg, et al. 2016a; Pradeep et al. 2017; Garg and Pradeep 2017; Kumari et al. 2016, 2017), four RSV studies (Pradeep et al. 2015; Pradeep, Garg, et al. 2016a; Garg and Pradeep 2017; Pankaj et al. 2018), and four SIM studies (Pradeep et al. 2012; Pradeep, Rao, et al. 2013; Pradeep and Thorat 2010; Rao et al. 2013) that reported complete 6‐month PPD and CAL data. Two multi‐arm trials contributed data to more than one statin subgroup, resulting in 14 datasets derived from 12 unique studies. In these multi‐arm trials, the sample size of the shared placebo control group was evenly split across the relevant comparison arms, while means and standard deviations were kept unchanged, in order to avoid double‐counting participants and to comply with Cochrane recommendations for handling shared control groups. Accordingly, three independent 6‐month meta‐analyses were performed for ATV, RSV, and SIM.

##### 3.7.1.1 ATV (6 Months)

Six studies were included. The random‐effects model showed a significant PPD reduction favoring ATV (MD: 1.39 mm, 95% CI: 0.99–1.79, *p* < 0.0001; *I*² = 86.5%). For CAL, the pooled effect also favored ATV (MD: 1.70 mm, 95% CI: 1.22–2.19, *p* < 0.0001; *I*² = 88.4%).

##### 3.7.1.2 RSV (6 Months)

Four studies contributed data. RSV produced a larger PPD reduction than ATV (MD: 1.91 mm, 95% CI: 1.35–2.48, *p* < 0.0001; *I*² = 96.4%), while SIM showed the largest pooled PPD effect. CAL gain was also significant (MD: 1.94 mm, 95% CI: 1.33–2.56, *p* < 0.0001; *I*² = 97.5%).

##### 3.7.1.3 SIM (6 Months)

Four studies were included. SIM significantly improved PPD (MD: 2.34 mm, 95% CI: 1.65–3.03, *p* < 0.0001; *I*² = 78.5%) and CAL (MD: 2.17 mm, 95% CI: 1.68–2.66, *p* < 0.0001; *I*² = 55.0%).

Overall, across all statins, the meta‐analysis shows a consistent benefit on average in both PPD and CAL at 6 months, with effect sizes often exceeding 1 mm; however, these values should be interpreted cautiously due to substantial heterogeneity. The magnitude of CAL and PPD improvement was greatest for SIM, followed by RSV and ATV. Numerically larger pooled MDs were observed for SIM; however, no head‐to‐head comparisons were available.

These pooled estimates should be interpreted with caution because between‐study heterogeneity was very high (*I*² values frequently > 80%), reflecting differences in study design, patient characteristics, and defect morphology. Therefore, the direction of effect appears consistent across the included trials, but the precise magnitude of the additional benefit remains uncertain.

#### 3.7.2 Surgical Therapy (9‐Month Meta‐Analysis)

Figure 4 illustrates the pooled 9‐month outcomes for the three surgical studies, showing MDs for both PPD and CAL. The forest plots display individual study effects, confidence intervals, and study weights, enabling a clear visualization of the overall benefit and the moderate heterogeneity observed across trials. The pooled dataset for Figure 4 was derived from three surgical studies reporting complete 9‐month PPD and CAL data (Pradeep, Garg, et al. 2016b; Pradeep, Karvekar, et al. 2016; Martande et al. 2016).

Of the six surgical studies, three provided numerical data for PPD and CAL at 9 months, enabling quantitative synthesis. Notably, the 9‐month pooled analyses included only Step 3 surgical trials; Step 4/SPC maintenance trials were not pooled and were synthesized narratively.

##### 3.7.2.1 PPD (9 Months)

The pooled analysis demonstrated a significant benefit favoring statins (MD: 0.80 mm, 95% CI: 0.56–1.05, *p* < 0.0001; *I*² = 43.4%), indicating moderate heterogeneity.

##### 3.7.2.2 CAL (9 Months)

Statins also produced a significant CAL gain at 9 months (MD: 0.69 mm, 95% CI: 0.42–0.96, *p* < 0.0001; *I*² = 34.7%).

Overall, compared with non‐surgical therapy, the effect sizes in surgical statin trials were smaller, generally 0.6–0.8 mm, but remained statistically significant and may be clinically relevant.

#### 3.7.3 Pre‐Implant Bone Regeneration Procedures

The two studies on pre‐implant bone regeneration procedures lacked compatible numerical datasets for pooling; therefore, results were narratively synthesized. Cruz et al. demonstrated significantly reduced horizontal ridge width loss and vertical dimensional change at 3 months in the SIM group compared with the control (Cruz et al. 2021). In contrast, Yaghobee et al. did not evaluate horizontal or vertical ridge dimensional changes but instead performed histologic and histomorphometric analyses at 9 months (Yaghobee et al. 2020).

In the latter study, both groups showed comparable amounts of newly formed bone and residual graft material, indicating no statistically significant benefit from adjunctive SIM.

## 4 Discussion

This systematic review and meta‐analysis evaluated the adjunctive use of locally delivered statins in non‐surgical and surgical periodontal therapy, as well as in pre‐implant bone regeneration procedures. Overall, the findings indicate that locally applied statins appear to provide clinically meaningful adjunctive improvements in PPD and CAL when used with NSPT. In surgical therapy, the benefits are more modest, yet still significant, whereas evidence for pre‐implant bone regeneration applications remains limited and heterogeneous.

In non‐surgical treatment, pooled 6‐month data showed additional PPD reductions of approximately 1.4–2.3 mm and CAL gains of 1.7–2.2 mm compared with SRP alone. Although these effect sizes are relatively large for adjunctive periodontal therapy, most included trials enrolled sites with deep baseline periodontal pockets or defect‐associated lesions (e.g., intrabony or furcation defects), where greater absolute improvements are expected following therapy. Therefore, these pooled estimates should be interpreted in the context of the included populations and are not broadly generalizable across all clinical scenarios. In surgical therapy, three RCTs contributed to 9‐month meta‐analyses, which demonstrated smaller but still clinically relevant improvements of about 0.7–0.8 mm in both PPD and CAL. Pre‐implant bone regeneration trials were too few and heterogeneous for quantitative synthesis and yielded inconsistent radiographic outcomes.

These results align with previous systematic reviews investigating the adjunctive use of statins in periodontal therapy. The recent review by Greethurst et al. (2024) reported overall beneficial effects of locally delivered statins on clinical and radiographic parameters, including improvements in PPD, CAL, and bone fill. In their meta‐analysis, clinical outcomes were pooled across statin types and periodontal interventions, and SIM demonstrated significantly greater PPD reduction than ATV at 6 and 9 months, although no differences between statins were observed for the remaining outcomes. However, this review did not differentiate between non‐surgical and surgical periodontal interventions when pooling clinical outcomes (Greethurst et al. 2024).

Earlier systematic reviews similarly reported beneficial effects of statins as adjuncts to periodontal therapy but largely focused on non‐surgical treatment. A systematic review evaluating host‐modulatory agents in conjunction with NSPT (Donos et al. 2020) reported that locally delivered statins significantly improved PPD reduction in infrabony defects compared with mechanical debridement alone. Another systematic review examining both local and systemic statin administration by Bertl et al. (2017) found that adjunctive local statins used with SRP resulted in significantly greater reductions in PPD, radiographic defect depth, and bleeding indices, as well as greater CAL gains, whereas systemic statin administration did not demonstrate comparable benefits. Similarly, a subsequent meta‐analysis evaluating statins as sole adjuncts to mechanical periodontal therapy by Muniz et al. (2018) reported significant improvements in PPD, CAL, and intrabony defect resolution compared with SRP alone, with SIM showing additional benefits across several clinical parameters.

Taken together, these previous reviews support the overall adjunctive benefit of statins in periodontal therapy, but primarily focused on non‐surgical treatment or comparisons between local and systemic statin delivery.

In contrast, the present review extends the available evidence by separately analyzing Step 2 NSPT and Step 3 surgical periodontal therapy, as well as incorporating pre‐implant bone regeneration procedures. This approach allows a more differentiated evaluation of treatment effects across periodontal treatment stages and highlights differences in the magnitude of benefit between non‐surgical and surgical interventions, thereby providing a more clinically meaningful interpretation of statin effects across treatment stages.

Importantly, current periodontal guidelines do not yet recommend statins as routine adjuncts (Herrera et al. 2022; Sanz et al. 2020); our findings suggest potential benefit but also highlight substantial heterogeneity and methodological limitations that currently preclude strong guideline‐level recommendations.

However, because heterogeneity in the non‐surgical meta‐analysis was extremely high (*I*² frequently exceeding 80%–90%), the pooled effect sizes should be interpreted with caution. This heterogeneity likely reflects variations in baseline disease severity, defect morphology, patient characteristics (e.g., smokers or individuals with diabetes), statin formulations, and adjunctive regenerative materials used across trials.

The findings consistently indicate a direction of benefit favoring statins, but the magnitude of the effect remains uncertain. Therefore, these results should be interpreted cautiously and not considered precise estimates of clinical efficacy.

Clinically, the available evidence primarily reflects sites with deeper baseline pockets and defect‐based lesions (e.g., intrabony or furcation sites) as reported in the included trials. Therefore, any potential benefit should be interpreted in that context and should not be extrapolated to all clinical scenarios. The following sections discuss Step 2 periodontal therapy (NSPT), Step 3 periodontal therapy (surgical procedures), and pre‐implant bone regeneration procedures in more detail, with emphasis on sources of heterogeneity and clinical applicability.

### 4.1 Step 2 Periodontal Therapy (Non‐Surgical Treatment)

Numerous RCTs support the adjunctive use of locally delivered statins with NSPT, consistently reporting greater improvements in PPD and CAL than SRP alone (Pradeep et al. 2012, 2015; Garg and Pradeep 2017).

Our 6‐month meta‐analysis confirmed this pattern, indicating additional PPD reductions of roughly 1.4–2.3 mm and CAL gains of 1.7–2.2 mm. Although effect sizes appear large, they likely reflect study populations with deep baseline pockets or intrabony/furcation defects, where greater absolute improvements are expected.

Although pooled estimates differed numerically between statins, no head‐to‐head comparisons or network meta‐analysis were performed; therefore, between‐statin comparisons remain indirect. Pooled estimates should be interpreted cautiously due to substantial heterogeneity (*I*² often > 80%), which may be influenced by differences in baseline disease severity, patient phenotypes (e.g., smokers or individuals with type 2 diabetes), defect morphology, statin formulations, and quality of mechanical therapy.

Baseline disease severity varied across studies and may partly explain the observed heterogeneity in pooled effect estimates. Defect morphology ranged from intrabony lesions to furcation defects, which respond differently to regenerative agents. Patient phenotypes also differed; some trials included smokers or individuals with type 2 diabetes, both of whom exhibit impaired periodontal healing and may show attenuated responses to statins. Baseline PPD and disease severity were inconsistent, and deeper initial pockets generally allow for larger numerical reductions, contributing to heterogeneity between studies. Finally, statin formulations were not standardized, with differences in concentration, vehicle composition, and viscosity that likely influenced local retention and release kinetics.

In addition, the quality and reporting of the underlying mechanical periodontal therapy varied between trials; although statins were consistently administered as adjuncts to conventional SRP performed according to standard clinical protocols, detailed information on operator experience, instrumentation methods, and objective measures of treatment quality was often incompletely reported, which may have further contributed to heterogeneity in treatment effects.

Most trials with extractable data originated from a limited number of research groups, particularly those associated with Pradeep et al. which may restrict the generalizability of the findings. Independent replication remains limited. Some evidence suggests that early improvements can be detected as soon as 45 days (Gunjiganur Vemanaradhya et al. 2017), but radiographic defect fill typically becomes apparent after 6 months (Pradeep, Garg, et al. 2016a; Pradeep et al. 2017; Rao et al. 2013), indicating that longer follow‐up is required to fully capture regenerative effects.

Reapplication of statin gel at 6 months did not confer additional benefit in the available RCTs (Pradeep, Garg, et al. 2016a; Garg and Pradeep 2017), suggesting that a single application at the time of SRP may be sufficient under current protocols. Overall, the direction of effect consistently favored statins, but the pooled MDs should be viewed as approximate indicators rather than precise predictions of effect magnitude.

### 4.2 Step 3 Periodontal Therapy (Surgical Treatment)

In Step 3 periodontal therapy (surgical treatment), locally delivered statins also appear to enhance the clinical outcomes, particularly when combined with biologically active adjuncts such as PRF, HA, EDTA, or barrier membranes. Our 9‐month meta‐analysis showed additional improvements of 0.80 mm in PPD and 0.69 mm in CAL compared with control procedures, with moderate heterogeneity (*I*² ≈ 35%–45%). These gains are smaller than those observed with non‐surgical therapy but still fall within a range that is often considered clinically relevant in trials of regenerative periodontal therapy.

Individual trials suggest that statins may have synergistic effects when integrated into regenerative protocols. For example, PRF combined with ATV or RSV improved defect fill in angular defects (Pradeep, Garg, et al. 2016b; Martande et al. 2016), and PRF + HA + RSV provided superior CAL and PPD outcomes compared with PRF + HA alone (Pradeep, Karvekar, et al. 2016). Similarly, root surface conditioning with EDTA before SIM application enhanced CAL and PPD improvements, and the combination of EDTA, SIM, and a PBM yielded the greatest reduction in defect depth (Issa et al. 2020). These findings are biologically plausible, given that PRF provides a fibrin‐based scaffold enriched with growth factors and cytokines that support tissue regeneration (Jamjoom 2024), while statins stimulate osteogenesis and angiogenesis (Tahamtan et al. 2020; Kommuri et al. 2025; Wang and Xu 2025).

Nevertheless, the number of surgical RCTs is small, and most come from a limited number of centers, which may inflate effect estimates and restrict external validity. More independent, multicentre trials with standardized protocols are needed before adjunctive statins can be recommended as routine components of regenerative periodontal surgery.

### 4.3 Pre‐Implant Bone Regeneration Procedures

Evidence for statin use in pre‐implant bone regenerative procedures remains limited and inconsistent. Two RCTs evaluated locally delivered SIM prior to implant placement (Cruz et al. 2021; Yaghobee et al. 2020). One trial reported significantly smaller horizontal ridge‐width loss and reduced vertical dimensional change in extraction sockets at 3 months (Cruz et al. 2021), whereas the sinus augmentation study by Yaghobee et al. found no significant between‐group differences in histologic and histomorphometric outcomes (e.g., mineralized tissue formation) when SIM was combined with bovine bone mineral (BBM) (Yaghobee et al. 2020).

These mixed findings, together with the small sample sizes and methodological heterogeneity, preclude firm conclusions regarding the benefit of statins in pre‐implant bone regeneration. Preclinical data suggesting improved osseointegration via upregulation of BMP‐2, promotion of osteoblast differentiation, and inhibition of osteoclast activity are promising (Tahamtan et al. 2020; Kommuri et al. 2025), but translation into predictable clinical benefits has yet to be demonstrated. At present, locally delivered statins for pre‐implant bone regeneration remain investigational and are not part of routine clinical practice.

Given the extremely limited number of trials and the substantial methodological heterogeneity between them, the current evidence base is insufficient to support any clinical recommendations. Furthermore, standardized outcomes specific to pre‐implant bone regeneration (e.g., ridge dimensions, graft maturation, and implant site‐development–related endpoints) are largely absent. Well‐designed, adequately powered RCTs with standardized defect types and harmonized radiographic and clinical outcome measures are essential before the adjunctive use of statins can be meaningfully evaluated in pre‐implant regenerative dentistry.

### 4.4 Limitations and Future Research

This review has several methodological limitations. First, some included RCTs were multi‐arm trials with two different statin intervention groups (e.g., an ATV arm and an RSV arm) sharing a single placebo control group. As the meta‐analysis was conducted separately for each statin type, these studies contributed more than one dataset. To avoid double‐counting participants, the shared control group was evenly split across comparisons, in line with Cochrane recommendations. However, the pooled effect estimates for each statin in multi‐arm trials remain approximations, because splitting the shared placebo group does not fully account for the correlation between the multiple intervention arms originating from the same study.

Second, substantial heterogeneity in the non‐surgical meta‐analysis means that pooled MDs primarily indicate the direction of effect rather than precise effect sizes.

Third, many included trials originated from a small number of research groups, which may limit generalizability and introduce publication bias. In particular, a large proportion of the extractable data, especially for non‐surgical and surgical trials, comes from a single research group (Pradeep et al.), which introduces a structural RoB arising from repeated methodologies, overlapping patient populations, and potentially similar operator effects. The overrepresentation of studies from a single research group reduces external validity and may lead to an overestimation of the consistency of treatment effects.

Fourth, variability in defect morphology, baseline disease severity, patient populations (including smokers and individuals with diabetes), statin formulations, and adjunctive materials reduces comparability between studies. In addition, the quality and reporting of the underlying mechanical periodontal therapy were inconsistently described, and objective measurements of instrumentation quality were rarely provided. These limitations reduce the ability to attribute observed treatment effects specifically to adjunctive statin therapy rather than differences in mechanical debridement.

Moreover, the simplified two‐level RoB grading (+/−) reflects the limited methodological reporting in many included studies, in which unclear‐risk domains could not be reliably differentiated from high‐risk assessments.

Taken together, these factors limit the certainty of the pooled estimates and warrant cautious interpretation. In addition, the small number of trials within each statin‐specific meta‐analysis precluded formal sensitivity analyses and prevented assessment of publication bias using funnel plots or Egger's regression testing. Therefore, small‐study effects cannot be excluded.

Given these limitations, future research should prioritize adequately powered, multicentre RCTs. These studies should include standardized reporting of periodontal and radiographic outcomes, longer follow‐up periods, and rigorous documentation of mechanical instrumentation protocols. They should also address the appropriate handling of multi‐arm designs. Direct head‐to‐head comparisons between different statin types and concentrations are needed, as well as studies in diverse patient populations and defect types. Such studies will clarify which clinical scenarios experience the greatest benefit from adjunctive statin therapy.

With regard to clinical applicability, findings should be interpreted in relation to baseline pocket depth, defect morphology, and patient‐related risk factors. Pooled estimates should not be used as effect‐size predictions applicable to all clinical scenarios.

Finally, none of the included trials performed formal cost‐effectiveness analyses. In addition, local statin formulations, compounding procedures, and delivery systems may vary across clinical settings and could influence overall treatment costs. Therefore, the economic implications of adjunctive local statin therapy remain uncertain.

## 5 Conclusions

Locally delivered statins were associated with improvements in clinical outcomes, particularly PPD and CAL, when used as adjuncts to NSPT. At 6 months, non‐surgical therapy in combination with statins provided additional improvements of roughly 1–2 mm in both PPD and CAL. Numerically larger pooled MDs were observed for SIM; however, no direct head‐to‐head comparisons were performed. These values should be regarded as approximate, given the high between‐study heterogeneity.

In surgical periodontal therapy, statins produced smaller but still significant additional gains of about 0.7–0.8 mm in PPD and CAL at 9 months, especially when combined with regenerative adjuncts such as EDTA, PBM, PRF, or HA.

For pre‐implant bone regeneration applications, current evidence is limited to two heterogeneous RCTs with inconsistent radiographic outcomes without long‐term data; therefore, no definitive recommendations can be made at this stage.

Overall, local statin therapy appears safe and well tolerated, but further high‐quality trials with longer follow‐up and standardized protocols are required before its routine clinical use can be recommended.

Future studies should aim to define the optimal statin type, dose, and delivery protocols and identify patient‐ and defect‐specific indications in which adjunctive local statin therapy provides a clinically meaningful advantage over conventional periodontal therapy and pre‐implant bone regeneration procedures alone.

## Author Contributions

F.A. conceived and designed the study. S.H. and N.M. performed the search strategy and extracted the study data. K.Y. extracted all numerical data used specifically for the meta‐analysis, which were cross‐checked by L.O. K.Y., S.H., N.M., L.O. and F.A. drafted the manuscript. K.Y. and F.A. critically revised the manuscript. All authors read and approved the final manuscript.

## Funding

The authors have nothing to report.

## Ethics Statement

This article is based on previously conducted studies and does not contain any studies with human participants or animals performed by any of the authors.

## Consent

The authors have nothing to report. This study is a systematic review and does not involve any new human data collection.

## Conflicts of Interest

The authors declare no conflicts of interest.

## Supporting information

Supporting Table S1

## Data Availability

The datasets generated and/or analyzed during the current study are available from the corresponding author on reasonable request.
