# Supplementary material for: Adjunctive Use of Locally Delivered Statins in Periodontal Therapy and Pre‐Implant Bone Regeneration: A Systematic Review and Meta‐Analysis
Source: Clin Exp Dent Res. 2026 Apr 28;12(2):e70364. doi: 10.1002/cre2.70364 (PMC13122124; doi:10.1002/cre2.70364)
Supplement: Supplementary file 1 — Supporting Table S1 [file CRE2-12-e70364-s001.docx]

**Adjunctive Use of Locally Delivered Statins in Periodontal Therapy and Pre-Implant Bone Regeneration: A Systematic Review and Meta-Analysis**

Koki Yoshida, Sofia Heller, Nur Mousa, Lovisa Olén, Farah Asa’ad

**Supplementary Table S1.** **Diagnostic criteria, defect characteristics, and baseline severity of included studies**

Eligibility criteria and baseline thresholds were recorded verbatim when available (e.g., minimum PD/CAL/defect depth); otherwise, baseline descriptive values were summarized in the corresponding study tables.

Diagnostic definitions, defect characteristics, and baseline severity were extracted directly from the Methods and baseline characteristics sections of the original full-text articles. Where exact baseline means were not consistently reported, inclusion criteria or approximate ranges described by the authors were summarized. No post hoc reclassification or recalculation of numerical values was performed. Where diagnostic thresholds were not explicitly stated, the study was classified based on the authors’ description of the population (e.g., chronic periodontitis; maintenance patients) and the defect type under investigation.

| **Study** | **Periodontitis definition / criteria (as reported)** | **Defect type / treated sites** | **Baseline severity (as reported)** |
| --- | --- | --- | --- |
| Garg & Pradeep 2017 | Chronic periodontitis (trial at patient/site level; furcation trial) | Class II furcation defects | Eligibility defined as Class II furcation defects; baseline clinical parameters reported in baseline tables. |
| Gunjiganur Vemanaradhya et al., 2017 | Chronic periodontitis | Periodontal pockets (NSPT sites) | Inclusion: pockets ≥5 mm (45-day follow-up study). |
| Kumari et al., 2016 (T2DM) | Chronic periodontitis + type 2 diabetes mellitus | NSPT sites (periodontal pockets) | Inclusion: PD ≥5 mm and CAL ≥3 mm (baseline criteria stated in eligibility). |
| Kumari et al., 2017 (smokers) | Chronic periodontitis in smokers | NSPT sites (periodontal pockets) | Inclusion: PD ≥5 mm or CAL ≥4 mm and vertical bone loss ≥3 mm; smoker population. |
| Pankaj et al., 2018 | Chronic periodontitis | Intrabony defects | Enrolled intrabony-defect sites; Eligibility included PD ≥5 mm; baseline values reported in original baseline tables. |
| Pradeep & Thorat, 2010 | Chronic periodontitis | NSPT sites (periodontal pockets) | Eligibility required PD 5–6 mm or CAL 4–6 mm; baseline values reported in baseline tables. |
| Pradeep et al., 2012 (SIM, furcation) | Chronic periodontitis | Class II furcation defects | Inclusion: PD ≥5 mm and vertical bone loss ≥3 mm in furcation area (eligibility). |
| Pradeep, Kumari, et al., 2013 | Chronic periodontitis | NSPT sites (periodontal pockets) | Eligibility required PD ≥5 mm and CAL ≥3 mm; baseline values reported in baseline tables. |
| Pradeep, Rao, et al., 2013 (T2DM) | Type 2 diabetes + chronic periodontitis | NSPT sites (periodontal pockets) | Eligibility required PD ≥5 mm; baseline values reported in baseline tables. |
| Pradeep et al., 2015 (RSV, NSPT) | Chronic periodontitis | NSPT sites (periodontal pockets) | Baseline PPD values reported in baseline tables. |
| Pradeep, Garg, et al., 2016 July | Chronic periodontitis with intrabony defects | Intrabony defects | Intrabony-defect sites; baseline clinical parameters reported in baseline tables. |
| Pradeep et al., 2017 (ATV vs ALN) | Chronic periodontitis | Intrabony-defect/periodontal defect sites (as per trial) | Eligibility defined as PD ≥5 mm and/or vertical bone loss ≥3 mm; baseline values reported in baseline tables. |
| Rao et al., 2013 (smokers) | Chronic periodontitis in smokers | NSPT sites (periodontal pockets) | Smoker population; eligibility defined as PD ≥5 mm (see extraction log). |
| Christiansen et al., 2023 | Periodontal maintenance patients (SPC/PMT population) | Maintenance sites / mini-flap wound healing | Baseline periodontal severity not the focus (maintenance-phase; staging reported but not used as baseline severity threshold). |
| Killeen et al., 2022 | Periodontal maintenance patients | Maintenance sites (post-therapy; locally applied SIM) | Baseline periodontal severity not the focus (maintenance-phase; staging reported but not used as baseline severity threshold). |
| Issa et al., 2020 | Chronic periodontitis | Angular intrabony defects (GTR context; membranes ± EDTA + SIM) | Inclusion focused on angular intrabony defects; defect-depth criterion reported (defect depth threshold stated in methods). |
| Martande et al., 2016 | Chronic periodontitis | Angular intrabony defects (surgery/OFD + PRF ± ATV) | Intrabony-defect sites; baseline clinical parameters reported in baseline tables. |
| Pradeep, Garg, et al., 2016 Dec | Chronic periodontitis | Intrabony defects (surgery/OFD + PRF ± RSV) | Intrabony-defect sites; baseline clinical parameters reported in baseline tables. |
| Pradeep, Karvekar, et al., 2016 | Chronic periodontitis | Mandibular Class II furcation defects (PRF + HA ± RSV) | Baseline: furcation-defect population; severity defined by furcation involvement + clinical parameters in baseline tables. |
| Cruz et al., 2021 | Not specified as periodontitis (extraction socket trial) | Extraction sockets | Periodontal baseline severity not applicable (socket healing endpoints). |
| Yaghobee et al., 2020 | Not specified as periodontitis (sinus augmentation trial) | Sinus augmentation sites | Periodontal baseline severity not applicable (histologic/histomorphometric outcomes). |

**Supplementary Appendix**

**Extraction Log for Supplementary Table S1**

(Eligibility criteria and baseline thresholds extracted verbatim from original Methods sections)

**Garg & Pradeep 2017 (Class II furcation; ATV/RSV)**

“Systemically healthy patients with mandibular Class II furcation defects and asymptomatic endodontically vital teeth were included.”

**Gunjiganur Vemanaradhya et al. 2017**

“Patients … with Probing pocket depth (PPD) of 4–6 mm and clinical attachment loss of 2–4 mm … were included.”

**Kumari et al. 2016 (T2DM; ATV)**

“Patients with well-controlled type 2 diabetes mellitus with probing depth (PD) ≥5 mm and clinical attachment level (CAL) ≥3 mm were included.”

Kumari et al. 2017 (smokers; ATV)

“Systemically healthy smokers with chronic periodontitis and sites with PD ≥5 mm or CAL ≥4 mm and vertical bone loss ≥3 mm were included.”

**Pankaj et al. 2018 (RSV vs metformin)**

“Chronic periodontitis patients with probing depth (PD) ≥5 mm and clinical attachment loss ≥3 mm were included.”

Pradeep & Thorat 2010 (SIM; chronic periodontitis)

“Patients with chronic periodontitis having pocket depth (PD) of 5–6 mm or clinical attachment loss (CAL) of 4–6 mm were included.”

**Pradeep et al. 2012 (SIM; furcation Class II)**

“Patients with buccal Class II furcation defects in endodontically vital mandibular molars were included.”

**Pradeep, Kumari, et al. 2013 (ATV; chronic periodontitis)**

“Systemically healthy individuals with PD ≥5 mm and clinical attachment level (CAL) ≥3 mm were included.”

**Pradeep, Rao, et al. 2013 (SIM; T2DM)**

“Patients with type 2 diabetes mellitus and chronic periodontitis with PD ≥5 mm were included.”

**Pradeep et al. 2015 (RSV; chronic periodontitis)**

“Systemically healthy patients with moderate periodontitis (probing depth 5–6 mm) were included.”

**Pradeep, Garg, et al. 2016 July (RSV vs ATV; intrabony)**

“Diagnosis of chronic periodontitis with probing depth (PD) ≥5 mm and clinical attachment level (CAL) ≥3 mm and angular bone loss ≥3 mm was required.”

**Pradeep et al. 2017 (ALN vs ATV)**

“Systemically healthy patients with deepest PD ≥5 mm or CAL 4–6 mm and vertical bone loss ≥3 mm were included.”

**Rao et al. 2013 (smokers; SIM)**

“Systemically healthy smokers with chronic periodontitis and sites having PD ≥5 mm or CAL ≥4 mm and vertical bone loss ≥3 mm were included.”

**Christiansen et al. 2023 (maintenance; SIM)**

“Subjects aged 40–85 years with Stage III–IV, Grade B periodontitis contributing one 6–9 mm periodontal pocket were included.”

**Killeen et al. 2022 (maintenance; SIM + EDTA)**

“Patients aged 40–85 years with Stage III, Grade B periodontitis contributing one interproximal 6–9 mm periodontal pocket were included.”

**Issa et al. 2020 (GTR; SIM; EDTA; membranes)**

“Patients with intrabony defects ≥3 mm in depth and probing depth ≥5 mm were included.”

**Martande et al. 2016 (OFD+PRF+ATV; intrabony)**

“Systemically healthy individuals with intrabony defects ≥3 mm in depth were included.”

**Pradeep, Garg, et al. 2016 Dec (PRF+RSV; intrabony)**

“Systemically healthy patients with intrabony defects and probing depth ≥5 mm were included.”

**Pradeep, Karvekar, et al. 2016 (furcation; RSV+PRF+HA)**

“Patients with mandibular Class II furcation defects were included.”

**Cruz et al. 2021 (extraction socket; SIM)**

“Subjects ≥18 years requiring posterior tooth extraction were included.”

**Yaghobee et al. 2020 (sinus augmentation; SIM+BBM)**

“Partially or fully edentulous patients requiring bilateral maxillary sinus augmentation were included.”
